# Topic Editorial on Flexible Electronics

**DOI:** 10.3390/mi15111350

**Published:** 2024-11-01

**Authors:** Meili Xia, Qiongfeng Shi

**Affiliations:** Interdisciplinary Research Center, School of Electronic Science and Engineering, Southeast University, Nanjing 211189, China; 230248948@seu.edu.cn

## 1. Introduction

**Figure 1:**
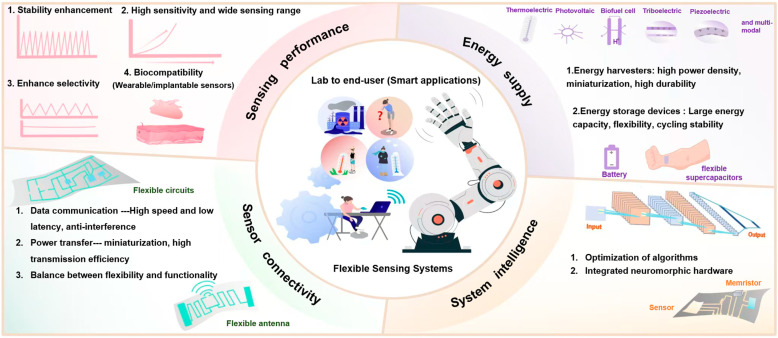
Challenges encountered by flexible electronics and systems in practical applications.

Fields such as the Internet of Things (IoT), smart healthcare, and intelligent manufacturing are at the forefront of technological advancement, involving the extensive deployment of numerous sophisticated electronic systems and devices [1,2,3,4]. Traditional rigid electronic devices are often limited by their inherent rigidity, restricted flexibility, and fragility, particularly in complex environments, such as wearable applications and advanced industrial contexts. In contrast, flexible electronics can conform to irregular surfaces, adapt to dynamic conditions, and withstand various mechanical deformations, thereby enabling reliable functionality across a diverse range of demanding settings [5,6]. This adaptability effectively addresses the increasingly varied requirements of modern electronic systems in both design and performance. Flexible sensors can operate effectively on dynamic and irregular surfaces, encompassing a wide range of sensing modalities, including pressure, temperature, humidity, biomarkers, metabolites, gasses, and more [7,8,9,10,11,12]. Furthermore, they hold significant potential for applications in health monitoring [13,14], neuroscience and biomedical studies [15], human–machine interfaces [16], and on-plant sensors for precision agriculture [17], among others. The future smart sensing system envisioned should operate autonomously, encompassing all aspects from stimulus detection and signal processing to data analysis and feedback. This system must not only facilitate sustained and self-sufficient operation but also demonstrate a high level of intelligence, enabling it to process substantial amounts of data and make intelligent decisions in real time. Despite significant advancements in laboratory research, flexible electronics continue to encounter various challenges in real-world applications [6,18], including performance enhancement, sensor connectivity (component interconnection/signal transfer), power supply, and system intelligence (Figure 1).

The theme of this Special Issue is “Flexible Electronics”, highlighting the latest advancements in the field. This edition comprises 13 published articles—4 research articles and 9 review articles—addressing key research areas such as sensing and substrate material design, the development of flexible antennas and circuits, and flexible energy storage and harvesting devices. By delving deeply into these essential subjects, this Special Issue aims to guide research efforts toward the common goals of enhancing the integration and intelligent development of flexible sensors, along with the creation of flexible smart systems with practical applications.

## 2. Overview of the Published Articles

To promote the practical application of flexible sensors, it is essential to integrate additional key components, including energy storage and functional devices, communication modules, and flexible circuits, while enhancing sensing computation capabilities through neuromorphic hardware to construct intelligent sensing systems. Experts in flexible electronics are actively working toward synergistic advancements in material design, system integration, and intelligent algorithm optimization, driving the transition of flexible smart systems from laboratory research to industrial applications. Flexible and stretchable pressure sensors are utilized to monitor human activities and biological signals, such as heartbeat, respiratory rate, and blood pressure, attracting significant attention for their potential integration into medical and healthcare devices. Thara Seesaard et al. [19] presented a comprehensive review of these sensors, covering fundamental principles, force/pressure-sensitive materials, fabrication techniques for low-cost, high-performance pressure sensors, investigations of sensing mechanisms (piezoresistivity, capacitance and piezoelectricity), and state-of-the-art applications. This review serves as a valuable resource for those seeking to understand flexible and stretchable pressure sensors. The authors emphasize that future research should focus on enhancing sensor performance, durability, and reliability, while ensuring biocompatibility and safety and addressing emerging trends. Key avenues for performance optimization include exploring new materials, optimizing sensor designs and architectures, the integration of multiple sensing mechanisms, and developing advanced signal-processing techniques. The study by Tingting Yu et al. [20] provides an exemplary approach to enhancing sensor performance through structural optimization. They developed a flexible piezoresistive sensor featuring a heterogeneous multi-material (HM) structure, incorporating a graphene foam (GF), a PDMS layer, and an interdigital electrode. This design achieved both high sensitivity (0.695 kPa^−1^) and an extensive measurement range (0–14,122 kPa) within a single device. Beyond structural advancements, optimizing performance through the development of new materials remains a central focus in current research. Flexible piezoelectric composites are distinguished by their high biocompatibility, flexibility, and sensitivity and excellent compatibility. Jinying Zhang et al. [21] reviewed the research progress of flexible piezoelectric composites, covering their types and typical fabrication techniques, and elaborating on their application value in underwater detection, e-skin sensing, wearable sensors, targeted therapy, and deep tissue ultrasound diagnosis. Additionally, their article summarizes the challenges faced by flexible piezoelectric composites, including non-toxicity and environmental sustainability, functional integration of sensing structures, and further optimization of performance trade-offs. This review provides valuable guidance for future research. In addition to optimizing single-sensor performance, multifunctional flexible sensors or multimodal flexible sensors capable of simultaneously sensing two or more external stimuli within a single unit have garnered significant attention. Ya Chang et al. [22] summarized recent efforts to fabricate high-performance multifunctional sensors, emphasizing functional materials, advanced structures, and intelligent systems, all of which enhance performance in terms of linearity, sensitivity, and detection diversity. They also identify several challenges, including the development of suitable nanomaterials, balancing sensitivity and functionality, and integrating with artificial intelligence systems. This review provides a comprehensive overview of multifunctional flexible sensors and offers valuable insights for future research and design. With regard to multimodal sensing, flexible image sensors that enable visual feedback of information are recognized as a research priority. The integration of stretchable displays with wearable sensors, actuators, and circuits enables the visualization of the collected bio-information to users [23], drawing significant attention to stretchable light-emitting diodes (LEDs) as a core component of these displays. Hamin Park et al. [24] provided a comprehensive summary of recent advancements in stretchable LEDs. Specifically, island–bridge, wavy buckling, and kirigami- or origami-inspired structures have been introduced to enhance strain distribution, though long-term durability remains a challenge. Intrinsically stretchable LEDs (is-LEDs) have been developed using electronic and optoelectronic materials, including conductive nanocomposites composed of one-dimensional (1D) nanomaterials and additive-blended conducting or semiconducting polymers. Nonetheless, issues related to performance and stability continue to limit their broader application. This review emphasizes the critical need for developing high-performance and durable is-LEDs, a key step toward advancing deformable display technology. Sensor research alone cannot meet specific application requirements, and thus a complete system must also integrate connectivity, signal acquisition, processing circuits, data analysis techniques, and other essential components to sensors. Thanh-Hai Nguyen et al. [25] developed a soft capacitive pressure sensor using spacer fabric, conductive inks, and encapsulation glue, achieving high sensitivity (0.04 kPa^−1^), a rapid recovery time (7 ms), and exceptional stability over 10,000 cycles. The sensor was integrated with conventional sensors and hardware components, enabling accurate recognition of human walking phases via a machine learning algorithm, achieving a high accuracy rate of 96%. This research highlights the critical role of advanced signal processing techniques and algorithms in the practical application of flexible sensors, as well as the importance of circuit and component flexibility.

Ambient energy harvesting technology is anticipated to enable sustainable, autonomous power generation, offering a continuous and reliable power source that reduces the need for frequent recharging and also suits energy-harvesting devices in flexible sensing systems. Related wearable energy harvesting technologies are being developed to support the development of portable and self-sustainable wearable electronics. Minki Kang et al. [26] reviewed the recent progress, potential, and technological challenges in energy harvesting technology (photovoltaic, biofuel cell, triboelectric, piezoelectric and thermoelectric) and accompanying technologies to construct a practical powering module, including power management and energy storage devices for the development of wearable devices. This review outlines a path forward to fully realize the potential of energy harvesters, leading to more versatile and autonomous wearable devices seamlessly integrating into daily life. In wearable energy harvesting, piezoelectric nanogenerators (PENGs) and triboelectric nanogenerators (TENGs) provide an efficient pathway for harvesting biomechanical energy and can be used as self-powered sensors. Among the PENGs and flexible piezoelectric sensors, the previously mentioned flexible piezoelectric composites are widely used [21]. Utilizing triboelectrification and electrostatic induction, dielectric polymers can be employed in TENG devices. To enhance the triboelectric properties, researchers have developed layer-structured dielectric films with high dielectric constants [27]. Minsoo P. Kim [28] reviewed the fundamental dielectric principles and applications of multilayered functional triboelectric polymers, including multilayer stacked dielectric polymers and functional polymer composites with high dielectric constant additives, demonstrating the potential of these materials in the development of highly sensitive multifunctional self-powered wearable devices. This review also outlines future research directions for multilayered dielectric materials that can enable the development of highly sensitive and multifunctional self-powered wearable devices. Polymer dielectric materials are extensively utilized in various emerging electronic devices, including memory devices and field-effect transistors (FET), due to their unique electrical, mechanical, and thermal properties. Wangmyung Choi et al. [29] systematically reviewed the advances in polymer dielectric materials, providing an in-depth summary of their applications in memory devices, FETs, and TENGs. They explored the synergistic interactions among devices based on polymer dielectric materials and their potential for integration, offering a comprehensive overview of their significant roles in advancing modern electronic technologies. This review offers new insights into the development of polymer dielectric materials and their applications in integrated systems with TENGs, FETs, and memory devices, serving as a crucial guide for advancements in flexible electronics, self-powered systems, and sustainable technologies. The efficiency of energy harvesting is very variable depending on the environment and energy sources. Power supplied by electrochemical energy storage devices (ESDs) tends to be more reliable than in situ energy harvesting. Commonly used ESDs for flexible sensors include lithium-ion batteries, zinc-ion batteries, and supercapacitors. Fiber supercapacitors, consisting of braidable yarns, are ideal for energy storage in flexible electronics and smart fabrics. Parya Teymoory et al. [30] investigated the energy and power densities, as well as the flexibility, of fiber supercapacitors, demonstrating that their power density satisfies the requirements of wearable devices and presenting a standardized technique for evaluating mechanical flexibility and stability. Their study also advises looking at new electrode-electrolyte composite designs and different methods of integrating fiber supercapacitors into textiles to balance flexibility and energy density. This paper offers valuable recommendations for using fiber-optic supercapacitors in wearable electronics, energy storage devices, and smart textiles. In addition to advancements in high-performance flexible energy harvesting and storage devices, power management units (PMUs) are equally essential. PMUs enhance the stability of the power supply by balancing energy input and output efficiently through algorithms and hardware, ensuring continuous power delivery to the sensing system while regulating power consumption. Simultaneously, efficient power transmission technologies are critical. The integration of these technologies enables more effective and sustainable energy management solutions.

Wired and wireless communication are essential components in constructing sensor systems, particularly within the biomedical field. Flexible antennas and functional circuits facilitate both power and data transmission, supporting efficient network operations. Wearable electronic devices equipped with flexible antennas and functional circuits enable the wireless, continuous, and real-time monitoring of human physiology and vital signs, offering a promising alternative to clinical diagnostics and postoperative treatment protocols. Minye Yang et al. [31] systematically reviewed the development of flexible antennas and circuits, covering both traditional/emerging materials, alongside distinctive system designs tailored for performance enhancement and specialized functionalities. They explored several prominent biomedical applications based on wearable electronic devices and emphasize that the development of chipless wearable electronics and an effective cross-body and in-body wireless power transfer system is crucial for advancing the development of epidermal and implantable sensors in the biomedical field. In the field of biomedical sensing, intraocular pressure (IOP) sensors serve as a prominent example of clinical utility, playing a critical role in the management of glaucoma. Kevin Y. Wu et al. [32] illuminated the critical role of IOP sensors in understanding and managing glaucoma, providing a detailed description of the design, preparation, performance, and applications of various types of IOP sensors. They also highlighted the potential and challenges associated with the use of wearable and implantable IOP sensors in glaucoma care. We contend that further optimization of the flexible antennas and circuits in these sensors, along with enhanced clinical applicability, could greatly expand their role in clinical applications. The performance of flexible antennas and circuits must be optimized from multiple perspectives, including material design, structural design, and processes. The substrate material plays a crucial role in the performance of high-performance flexible antennas, Praveen Kumar Sharma et al. [33] evaluated polydimethylsiloxane (PDMS) as a substrate for flexible/wearable antennas and sensors. This study demonstrates that the dielectric constant and loss tangent of PDMS is 6.2 and 25%, respectively, with *ε_par_* exceeding *ε_perp_* by 5.7%, indicating moderate anisotropy. Additionally, the dielectric constant decreases with increasing temperature, exhibiting anisotropic behavior. Furthermore, bending and anisotropy exert opposing effects on the resonance properties. These characteristics suggest that PDMS has the potential to serve as an ideal substrate material for flexible/wearable antennas and sensors.

## 3. Conclusions

The articles in this Special Issue highlight significant advances in the field of flexible electronics, providing a valuable resource for knowledge development. Despite rapid advancement in this domain, several challenges remain in realizing flexible smart systems for real-world applications. Optimizing the performance of flexible sensors is paramount, with an emphasis on enhancing stability and selectivity while achieving a balance between high sensitivity and a wide sensing range. Successful optimization depends on material design [34,35], structural improvements [36,37], process refinement [38,39], the integration of multiple sensing mechanisms [40,41], and the introduction of advanced signal processing techniques [42].

Smart sensing systems depend not only on sensors but also on connectivity, signal acquisition, processing circuits, and data analysis. To enable the continuous and self-sustainable operation of flexible sensing systems, current research should prioritize the development of flexible, stretchable, and miniaturized energy harvesting and storage devices with high energy conversion efficiency, as well as reliable and efficient wireless power transmission and power management systems [6]. Furthermore, advancing high-performance flexible antennas and circuits to ensure effective communication between sensors and control units, as well as secure connections between components, is a critical area for future exploration. Enhancing system intelligence is key to realizing diverse applications, which necessitates support from advanced data analysis algorithms, edge computing, and neuromorphic hardware [43,44]. Thus, the transition of flexible intelligent systems from laboratory settings to practical applications in the market and industry requires synergistic advancements across several fields, including materials science, electrical engineering, and information technology.

We hope that the articles in this Special Issue will stimulate readers’ interest in the latest advancements in flexible electronics and promote a renewed wave of research and exploration. As these critical domains continue to progress, we anticipate the imminent commercialization of a broader array of flexible smart sensors. Such advancements are expected to significantly enhance practical applications in the era of digitization and big data, ultimately improving the overall well-being of both industry and society. Importantly, this progress highlights the transformative potential of flexible electronics in addressing contemporary challenges.
